# circ_SEPT9, a newly identified circular RNA, promotes oral squamous cell carcinoma progression through miR‐1225/PKN2 axis

**DOI:** 10.1111/jcmm.15943

**Published:** 2020-10-14

**Authors:** Yilong Ai, Zhe Tang, Chen Zou, Haigang Wei, Siyuan Wu, Dahong Huang

**Affiliations:** ^1^ Foshan Stomatological Hospital School of Stomatology and Medicine Foshan University Foshan Guangdong China

**Keywords:** circ, miR‐1225, OSCC, PKN2, SEPT9, tumour progression

## Abstract

Circular RNAs (circRNAs) represent a newly discovered class of endogenous non‐coding RNAs which are widely expressed and play important roles in disease progression. However, the function of circRNAs in oral squamous cell carcinoma (OSCC) still remains largely unknown. In this research, we found that circ_SEPT9 was highly expressed in OSCC cell lines and tumour tissues. Results showed that circ_SEPT9 promoted OSCC proliferation and tumour growth. And, circ_SEPT9 also enhanced the migration and invasion of OSCC cells. Mechanically, we found that circ_SEPT9 acted as a sponge for miR‐1225 to rescue PKN2 expression in OSCC cells. Inhibition of circ_SEPT9/miR‐1225/PKN2 pathway could effectively block the proliferation and metastasis of OSCC cells. Our study provides strong evidence that circ_SEPT9/miR‐1225/PKN2 axis is a promising target for OSCC treatment.

## 1 INTRODUCTION

Oral squamous cell carcinoma (OSCC) ranks among the eighth most common cancer and accounts for 95% of the head and neck cancers.^1^ Although therapy has developed in the past decades, the survival of OSCC patients still remains very poor. The 5‐year survival rate of patients with metastasis is less than 40%, and most of the patients will die within 1 year.^2^ Investigation of the underlying molecular mechanism in OSCC metastasis is of great importance for diagnostic and therapeutic strategies.

Circular RNAs (circRNAs) represent a newly discovered class of endogenous non‐coding RNAs which are widely expressed in eukaryotic cells. With the rapid development of sequencing technology and bioinformatics, increasing numbers of non‐coding RNAs (ncRNAs) were identified. And, circRNAs are becoming more and more important in the research of ncRNAs.^3^, ^4^, ^5^ Circular RNAs are covalently closed loops without 5’ caps and 3’ poly‐A tails. Circular RNAs are resistant to RNase digestion because of its special structure.^4^, ^5^ Circular RNAs are generated from its precursor mRNA by non‐canonical splicing.^6^ Circular RNAs regulate the expression of gene mainly by interactions with microRNAs or competing with other endogenous RNAs in various biological activities.^7^ Increasing studies have shown that circular RNAs play critical roles in disease development and progression, particular in cancers.^8^, ^9^

Up to now, the function of circRNAs in OSCC still remains largely unclear. Recent studies with circular RNAs assay showed that circ_ SEPT9 (hsa_circ_0005320) which derived from SEPT9 was greatly up‐regulated in oral mucosal melanoma (OMM) primary tumour compared to paired adjacent normal tissues.^10^ circ_ SEPT9 located on chr17:75398140‐75398785 and 645bp in length. It has been identified to widely express in various cancer cell lines such as HepG2, K562, A549, McF7 and SY5Y indicating the important role of circ_SEPT9 in cancer progression. However, the expression and biological function of circ_SEPT9 urgently need further study in OSCC.

In our research, we elucidated that high expression of circ_SEPT9 contributed to the tumour proliferation and metastasis in OSCC. Furthermore, we demonstrated that hsa_circ_0005320 (circ_SEPT9) could act as sponge for miR‐1225 which could regulate PKN2 expression. Thus, we suggest that hsa_circ_SEPT9/miR‐1225/PKN2 signalling pathway is a promising target for OSCC therapeutic treatment.

## 2 MATERIALS AND METHODS

### 2.1 Cells culture

HN4, SCC‐9, SCC‐25, CAL‐27, SCC‐15, HSC‐3, UM1 and human oral keratinocyte (HOK) cells were obtained from Cell Bank of the Chinese Academy of Science (Shanghai, China) and American Type Culture Collection (Manassas, VA, USA). All cell lines were cultured under the introduction of the manufacturer.

### 2.2 Patient samples

A total of 15 paired OSCC and adjacent normal tissues were collected from Foshan Stomatological Hospital. All samples were collected under the regulation of the Ethics Committee of Foshan Stomatological Hospital, School of Stomatology and Medicine (ECR‐20200102). All patients have been informed the use of tissues and consent to the experiments.

The tissues were stored in −80°C until used. All patients received no preoperative anti‐cancer treatment.

### 2.3 RNA isolation and quantitative real‐time PCR

The RNA was extracted from patient samples and oral squamous cell carcinoma lines with the TRIzol kit (Invitrogen, Carlsbad, CA, USA) under the manufacturer's instructions. To deplete the linear RNA, the total RNA was treated with 2 U/ug RNase R for 45 min at 37°C (Epicentre Technologies, RNR07250). We used RNeasy Cleaning Kit (Qiagen) to purify the RNA. We performed the RT‐PCR of mRNA and circRNA by PrimeScript RT Reagent Kit (TaKaRa, Dalian, China). miRNA RT reaction was carried out by the miRNA First Strand Synthesis Kit (TaKaRa). Quantitative real‐time PCR (qRT‐PCR) was carried out by ABI system. The gene expression was analysed with 2^ΔΔCt^ method. Primers are as follows: circ_SEPT9 up: 5′‐CTGTGGCTGAGGCTACACC‐3′, down: 5′‐ACAGTGGCTCGGAGTAGGG‐3′; linear SEPT9 up: 5′‐TTCGGCTACGTGGGGATTG‐3′, down: 5′‐CTGCCCGACCACCATGATG‐3′; PKN2 up: 5′‐TGACCCTCGTTGTTCTACTAGC‐3′, down 5′‐GTTTCCGATCCTTTGAAGATCCA‐3′; and actin: 5′‐GAATCAATGCAAGTTCGGTTCC‐3′, 5′‐TCATCTCCGCTATTAGCTCCG‐3′.

### 2.4 Western blot

Cells were treated with protein lysis buffer (Beyotime, Shanghai, China), and the concentration of protein was identified by BCA Protein Assay (Solarbio, Beijing, China). 20 µg protein was separated by SDS‐PAGE and then transferred to PVDF membranes. The PVDF membrane was blocked with 5% milk for 2 hours at room temperature and incubated with antibodies for indicated proteins at 4°C overnight. The membrane was incubated with horseradish peroxidase (HRP)–conjugated secondary antibodies for 1 hour at room temperature. The bands were identified by chemiluminescence kit (Millipore Corporation, Billerica, MA, USA).

### 2.5 CCK8 Assay

Cell growth was identified by CCK8 assay (Cell Counting Kit‐8, Dojindo Molecular Technologies, Kumamoto, Japan). 5000 oral squamous cell carcinoma cells were seeded per well in 96‐well plates after transfections. At indicated time, 10 µL CCK8 reagents were added to the wells and incubated for 2 hours at 37°C. The absorbance at 450 nm was detected.^11^

### 2.6 Plasmids and transfection

Human circ_SEPT9 was synthesized and cloned into pcDNA3.1 vector by Geneseed Co., Ltd. (Guangzhou, China). Then, we transfected the oral squamous cell carcinoma lines cell lines with the plasmids and selected with G418.

### 2.7 Cell invasion assays

For the transwell assay, 1 × 10^5^ cells were suspended in serum‐free RPMI‐1640 medium and placed in the upper chamber which had been coated with matrigel (BD Biosciences, San Joses, CA, USA), while the complete RPMI‐1640 medium with 5% FBS was placed into the lower chamber. The transwell assays were incubated in 37°C for 48 hours. We removed the cells on the upper membrane and fixed the cells on the lower surface of the membrane with 4% PFA. Then, the membrane was stained with crystal violet and photographed by microscope.

### 2.8 Xenografts in nude mice

4‐ to 6‐week‐old BALB/c nude mice were obtained from Shanghai SLAC Laboratory Animal, Co., Ltd. (Shanghai, China). The nude mice were randomly divided into two groups. 2.5 × 10^6^ cells were subcutaneously injected into the flank of the nude mice. Tumour size was measured every three days. The volume of the tumour was calculated as: Volume = (length × width ^2^)/2. 36 days after the inoculation, the mice were killed. The tumour was removed and photographed. The size and weight of the tumour were recorded. All the animal experiments were approved by the Ethics Committee of Animal of School of Stomatology and Medicine, Foshan University.

### 2.9 Luciferase reporter assay

PKN2 3’ UTR was synthesized and inserted into pGL3 plasmids by Gene‐Great company (Wuhan, China). Then, we transfected the pGL3‐basic‐PKN2 and pGL3‐Renal into oral squamous cells by Lipofectamine 2000. The cells were transfected with miR‐1225 mimics, inhibitor or circ_SEPT9 by Lipofectamine 2000. The activity of luciferase was detected after 48 hours. Luciferase activity was normalized to activity of Renilla.

### 2.10 Bioinformatics

We used Circinteractome database to predict circ_SEPT9 potential target miRNA. We applied TargetScan to search microRNA target genes.

### 2.11 Statistical analysis

All data were showed as mean ± SD. All results were analysed by GraphPad Prism (GraphPad Software, La Jolla, CA, USA).^12^ The significance of differences was estimated by Student's *t* test or one‐way analysis of variance. The value of *P* < .05 was identified as statistically significant. *P < .05, **P < .01, ***P < .001.

## 3 RESULTS

### 3.1 Validation of circ_ SEPT9 expression in OSCC

**FIGURE 1:**
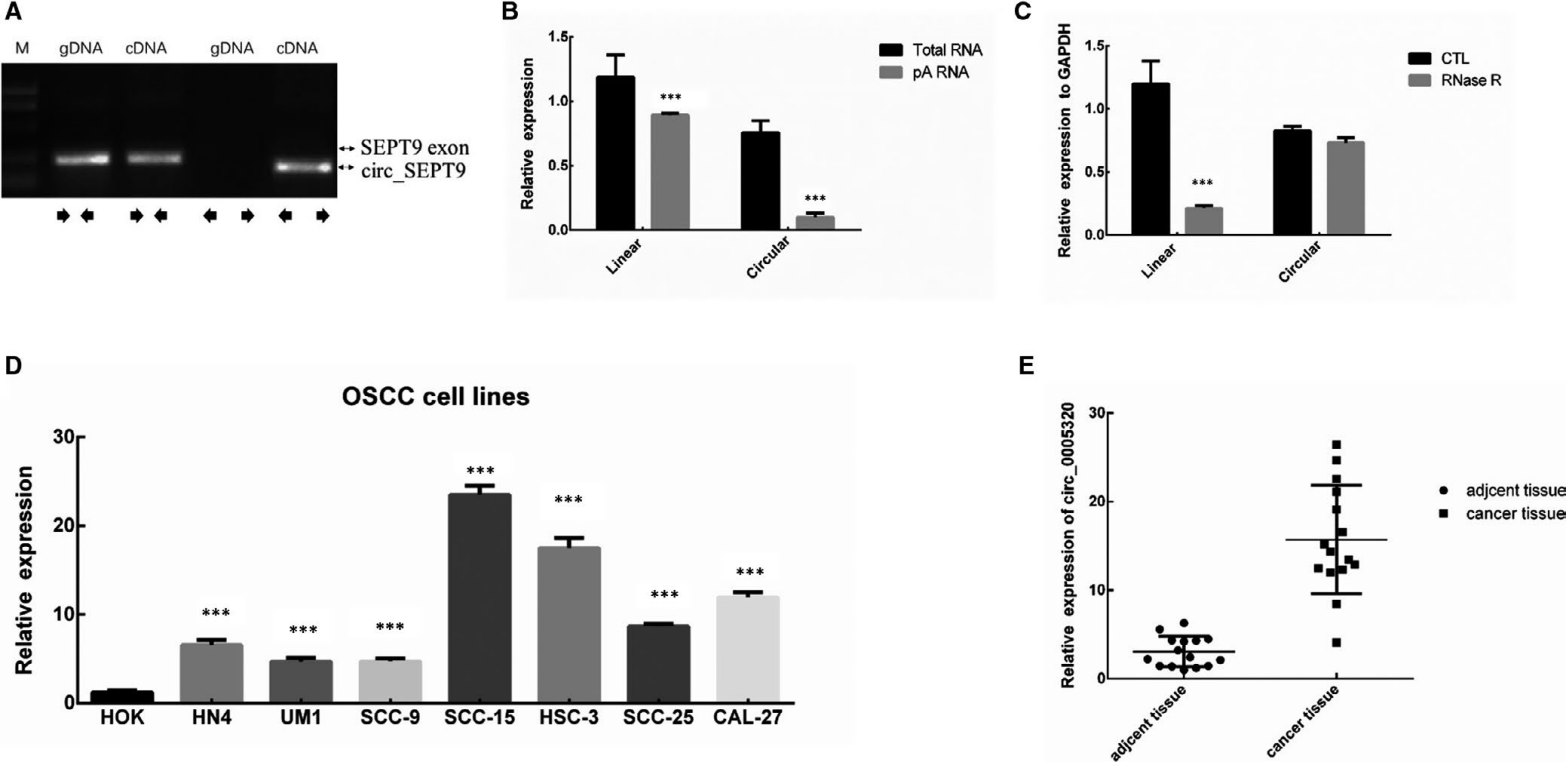
circ_SEPT9 is up‐regulated in oral squamous cell carcinoma. A, Special primers were designed to detect the linear and circular SEPT9 in gDNA and cDNA by reverse transcript PCR. B, OligodT and random primers were used in quantitative real‐time PCR (qRT‐PCR) in SCC‐15 cell line. circ_SEPT9 was absent in poly(A) enriched RNAs. C, qRT‐PCR was performed with RNase R treatment. Circular RNA was resistant to RNase R treatment. D, qRT‐PCR analysis was performed to detect the expression of circ_SEPT9 in OSCC and normal cell line HOK. E, qRT‐PCR analysis was performed to detect the expression of circ_SEPT9 in OSCC tissue and the adjacent normal tissue

Previous study reported that hsa_circ_ SEPT9 derived from SEPT9 was greatly up‐regulated in OMM tumour tissue compared to adjacent normal tissues by microassays.^10^ To validate the expression of circ_ SEPT9 in OSCC cells, we used convergent and divergent primers to perform PCR in genomic DNA (gDNA) and complementary DNA (cDNA) separately. Two sets of different primers were designed for PCR: Divergent primers were used to detect the circular SEPT9 RNA, and convergent primers were designed to amplify SEPT9 exon. As Figure 1A shows, convergent primers can amplify DNA from both gDNA and cDNA, while circ_ SEPT9 can only be amplified with divergent primers when using cDNA as template. Further experiments showed that the linear SEPT9 exon RNA expression level showed no difference between poly(A) enriched RNA and the total RNA in SCC‐15 cell line, while circular SEPT9 decreased obviously in poly(A) enriched RNA compared to the total RNA in SCC‐15 cells due to the lack of poly(A) (Figure 1B). To validate the circular characteristics of circ_ SEPT9, we performed the PCR experiments by pre‐treatment with RNase R which was a highly processive 3′ to 5′ exoribonuclease. As expected, the circular SEPT9 was more resistant to RNase R treatment than linear form (sixfold in SCC‐15 cells) (Figure 1C).

### 3.2 OSCC cells have higher expression of circ_SEPT9 than normal cells

To further investigate the roles of circ_SEPT9 in OSCC, we collected OSCC patients tissue, adjacent normal tissue, OSCC cell lines ( HN4, SCC‐9, SCC‐25, CAL‐27, SCC‐15, HSC‐3, UM1) and normal human oral keratinocyte cells (HOK). We performed qRT‐PCR to determine the expression level of circ_SEPT9 in OSCC cells and normal cells. The results showed that OSCC cells including HN4, SCC‐9,SCC‐25, CAL‐27, SCC‐15, HSC‐3, and UM1 had higher expression of circ_SEPT9 than HOK cells (Figure 1D). What's more, we also carried out qRT‐PCR in OSCC tissue and adjacent normal tissue. The results showed that cancer tissue had higher expression compared to the adjacent tissue (Figure 1E).

### 3.3 circ_SEPT9 does not influence SEPT9 expression

It has been reported that circRNAs may regulate cancer progression by competing with the splicing of pre‐mRNA, which would affect mRNA maturation and protein expression.^13^

**FIGURE 2:**
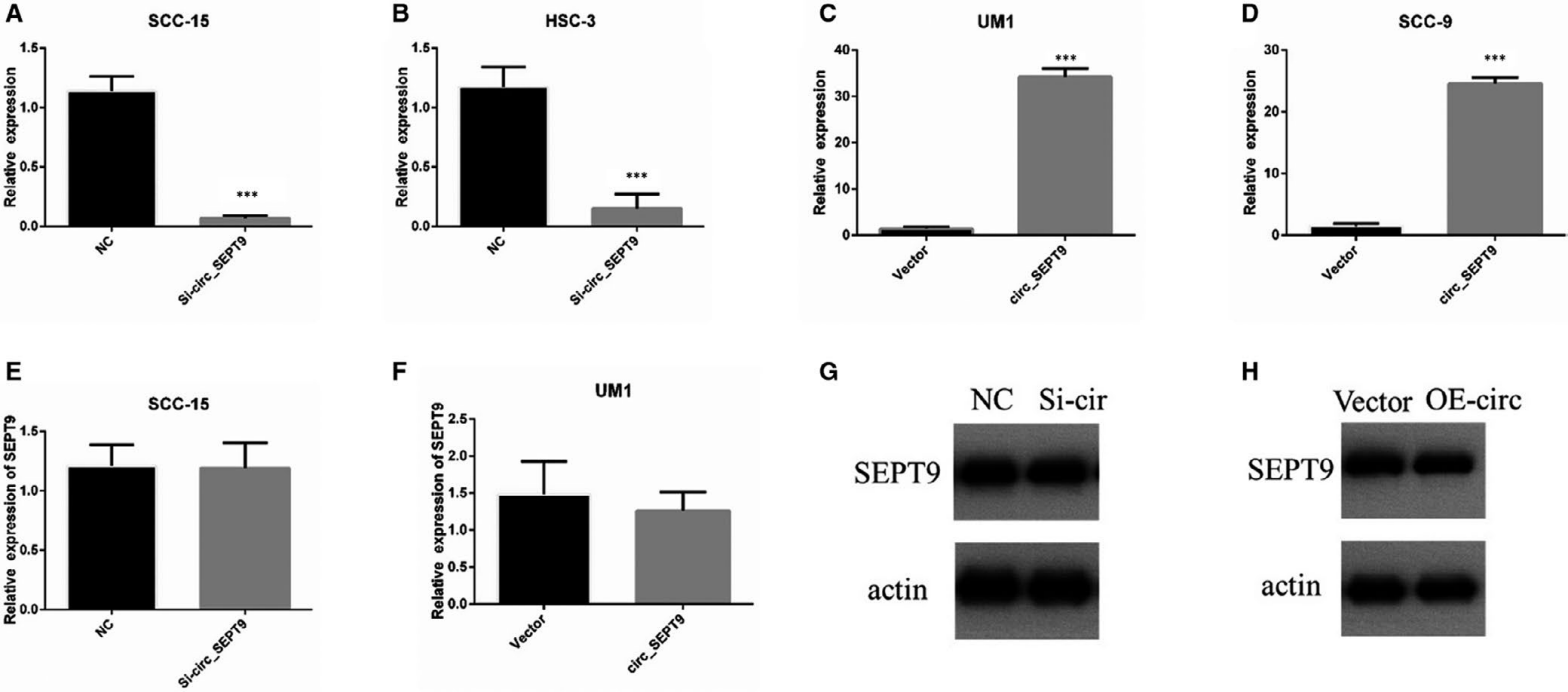
circ_SEPT9 does not influence SEPT9 mRNA and protein level. A‐D, The relative RNA level of circ_SEPT9 was detected by qRT‐PCR in SCC‐15, HSC‐3, UM1 and SCC‐9. E, The relative RNA level of linear SEPT9 was detected by qRT‐PCR in SCC‐15. F, The relative expression of linear SEPT9 was detected by qRT‐PCR in UM1. G, Western blot was used to detect SEPT9 protein level in SCC‐15 NC and si‐circ_SEPT9 cells. H, Western blot was used to detect SEPT9 protein level in UM1 vector and circ_SEPT9 cells

In order to explore the biological functions of circ_SEPT9 in OSCC, we firstly identified whether circ_SEPT9 could influence the transcription of SEPT9 in OSCC cells. We transfected siRNAs to knock down circ_SEPT9 expression in SCC‐15 (Figure 2A) and HSC‐3 (Figure 2B), and overexpression circ_SEPT9 in UM1 (Figure 2C) and SCC‐9 (Figure 2D). Firstly, we performed the qRT‐PCR to detect that whether circ_SEPT9 could influence the mRNA level of SEPT9 in OSCC cells. The qRT‐PCR results showed that circ_SEPT9 did not influence the mRNA level of SEPT9 in SCC‐15 cells (Figure 2E). Western blot results also confirmed that circ_SEPT9 did not influence the SEPT9 protein level (Figure 2G). We also overexpressed circ_SEPT9 in UM1 cells. Overexpression of circ_SEPT9 did not regulate mRNA and protein level of SEPT9 cells (Figure 2F,H). All the Western blot results have been quantified and analysed by ImageJ (Figure S1 A,B). As indicated, neither silence nor overexpression of circ_SEPT9 did not influence the mRNA and protein level of SEPT9 (Figure 2E‐H). All the results showed that circ_SEPT9 expression level did not effect SEPT9 linear mRNA and protein level.

### 3.4 circ_SEPT9 promotes proliferation and metastasis in OSCC

**FIGURE 3:**
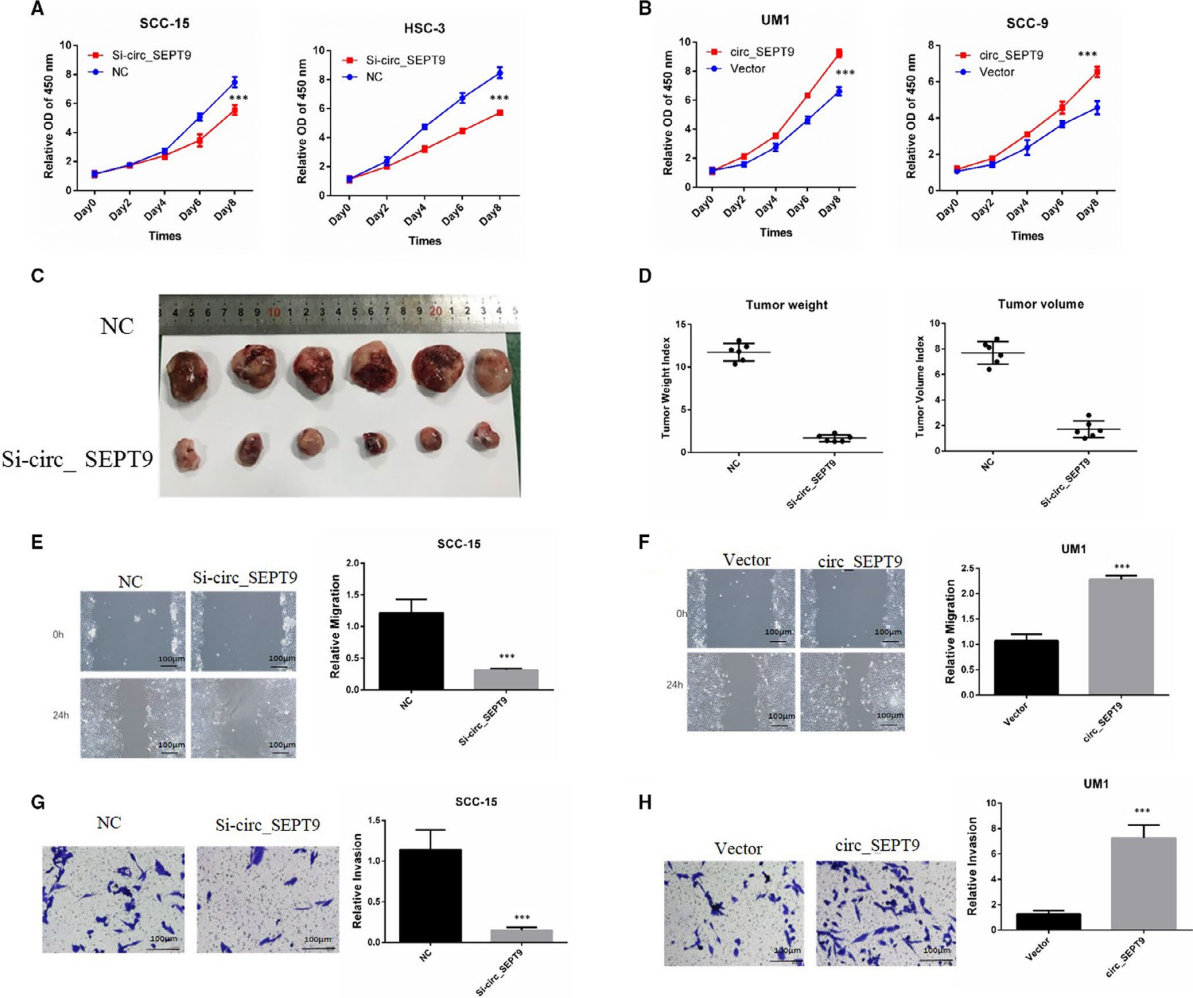
circ_SEPT9 promotes the progression of OSCC cells. A, CCK8 was performed to detect the growth rates of SCC‐15 and HSC‐3 with transfection of si_circSEPT9. B, CCK8 was used to detect the growth rates of UM1 and SCC‐9 with transfection of circ_SEPT9 overexpression plasmids. C, 2.5 × 10^6^ si‐circ_SEPT9 and NC SCC‐15 cells were subcutaneously injected into the flank of the nude mice (n = 6). Photographs of tumours derived from nude mice were shown. D, Tumour weight and tumour volume were analysed and shown. E, Wound assay was performed in SCC‐15 cells. Photographs were taken by microscope at 0 and 24 h. The scale bar is 100 μm. F, Wound assay was performed in UM1 cells. Photographs were taken by microscope at 0 and 24 h. The scale bar is 100 μm. G, Transwell assay was performed in SCC‐15 cells. Invasive cells were stained and counted. The scale bar is 100 μm. H, Transwell assay was performed in UM1 cells. Invasive cells were stained and counted. The scale bar is 100 μm

We detected the influence of circ_SEPT9 on proliferation and metastasis at first. Cell proliferation was measured by the CCK8 assay. Silencing of circ_SEPT9 significantly inhibited proliferation of SCC‐15 and HSC‐3 cells (Figure 3A). Overexpression of circ_SEPT9 promoted proliferation of SSC‐9 and UM1 cells (Figure 3B). In vivo experiments showed silence of circ_SEPT9 inhibited the tumour growth of SCC‐15 in nude mice (Figure 3C,D).

Moreover, wound assay and invasion assay demonstrated that silence of circ_SEPT9 markedly impeded the migration and invasion of SCC‐15 cells (Figure 3E,G). Overexpression of circ_SEPT9 promoted the migration and invasion of UM1 cells (Figure 3F,H). These data collectively indicated that circ_SEPT9 silence could retard the progression of OSCC cells, while overexpression of circ_SEPT9 could promote the progression.

### 3.5 circ_SEPT9 acts as the sponge for miR‐1225 in OSCC cell lines

Many researches showed that circRNAs could act as microRNA sponges and regulate the expression of microRNA.^14^ Our hypothesis is that circ_SEPT9 promotes the OSCC progression through functioning as microRNA sponge. We used circular RNA database (https://circinteractome.nia.nih.gov/) to search for the potential targeted microRNAs for circ_SEPT9. The database predicted the possible binding site between miR‐1225 and circ_SEPT9.

**FIGURE 4:**
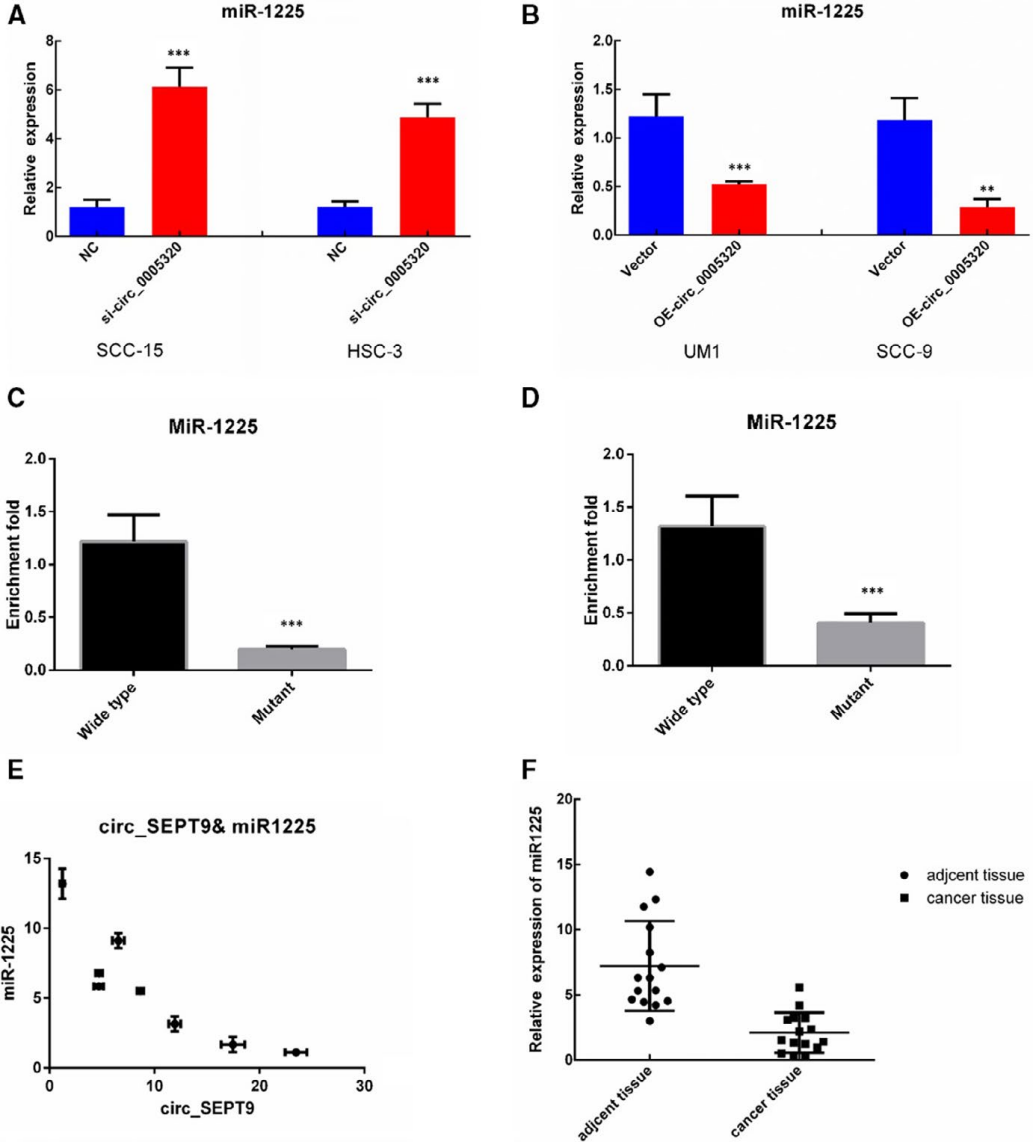
circ_SEPT9 acts as sponges for miR‐1225. A, Level of miR‐1225 was detected after silence of circ_SEPT9 in SCC‐15 and HSC‐3 cells. B, Level of miR‐1225 was detected after overexpression of circ_SEPT9 in SCC‐9 and UM1 cells. C, Pull down of circ_SEPT9 by biotin‐labelled wide‐type or binding site–mutated miR‐1225 in SCC‐15 cell lysis. D, Pull down of circ_SEPT9 by biotin‐labelled wide‐type or binding site–mutated miR‐1225 in UM1 cell lysis. E, Negative correlation between circ_SEPT9 and miR‐1225 in OSCC specimens detected by qRT‐PCR. F, qRT‐PCR analysis for the expression of miR‐1225 in OSCC cancer tissue and the adjacent normal tissue

To investigate the relationship between circ_SEPT9 and miR‐1225, we performed the following experiments. First, we detected whether circ_SEPT9 could influence miR‐1225 expression. We transfected OSCC cells with circ_SEPT9 overexpressing plasmids or siRNA targeted circ_SEPT9 and then used qRT‐PCR to detect the expression of miR‐1225. Results showed that silence of circ_SEPT9 increased the miR‐1225 expression (Figure 4A), while overexpression of circ_SEPT9 significantly decreased the expression of miR‐1225 (Figure 4B).

Furthermore, we used RNA pull‐down experiments to investigate the direct interaction between circ_SEPT9 and miR‐1225. Wide‐type and binding site–mutated miR‐1225 were labelled with biotin. As indicated, RNA pull‐down experiments showed the direct interaction between circ_SEPT9 and wide‐type miR‐1225 in SCC‐15 (Figure 4C) and UM1 (Figure 4D). And mutant of binding site significantly abrogated the interaction between circ_SEPT9 and miR‐1225(Figure 4C,D).

Interestingly, miR‐1225 expression was significantly lower in OSCC cells which had higher expression of circ_SEPT9. The expression of miR‐1225 was negatively correlated with the expression of circ_SEPT9（Figure 4E）. What's more, tumour tissue had lower expression of miR‐1225 than the compared adjacent normal tissue (Figure 4F). All the experiments above confirmed that circ_SEPT9 acted as microRNA sponge for miR‐1225.

### 3.6 miR‐1225 inhibits OSCC proliferation and metastasis

**FIGURE 5:**
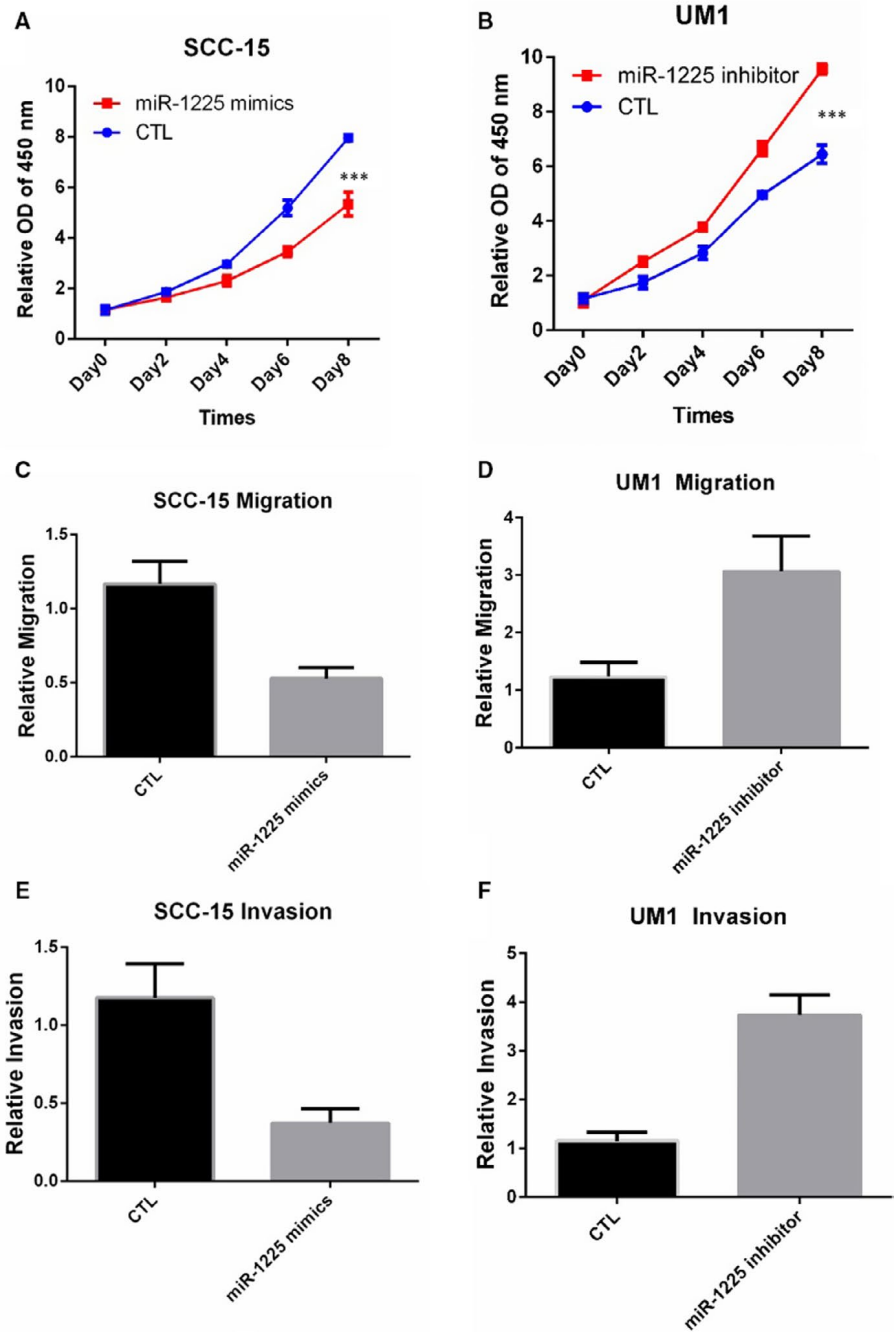
miR‐1225 promotes the proliferation, migration and invasion in OSCC. A, CCK8 was performed to detect the growth rate of SCC‐15 with transfection of miR‐1225 mimics. B, CCK8 was performed to detect the growth rate of UM1 with transfection of miR‐1225 inhibitor. C, Wound assay was performed in SCC‐15 cells. Photographs were taken by microscope at 0 and 24 h. D, Wound assay was performed in UM1 cells. Photographs were taken by microscope at 0 and 24 h. E, Transwell assay was performed in SCC‐15 cells. F, Transwell assay was performed in UM1 cells

As the above results showed that miR‐1225 was the target of circ_SEPT9, we wanted to know whether miR‐1225 was responsible for the proliferation and metastasis of OSCC cells. The function of miR‐1225 in OSCC was examined by CCK8 assay, wound assay and transwell assay. Overexpression of miR‐1225 by transfection of mimics decreased the growth rate of SCC‐15 (Figure 5A), and inhibition of miR‐1225 significantly enhanced the growth rate of UM1 (Figure 5B). Overexpression of miR‐1225 receded the migration and invasion ability of SCC‐15 (Figure 5C,E). What's more, miR‐1225 inhibition promoted the migration and invasion of UM1 (Figure 5D,F). Thus, these results suggested that miR‐1225 contributed to the progression of OSCC.

### 3.7 circ_SEPT9 regulates PKN2 expression through miR‐1225

**FIGURE 6:**
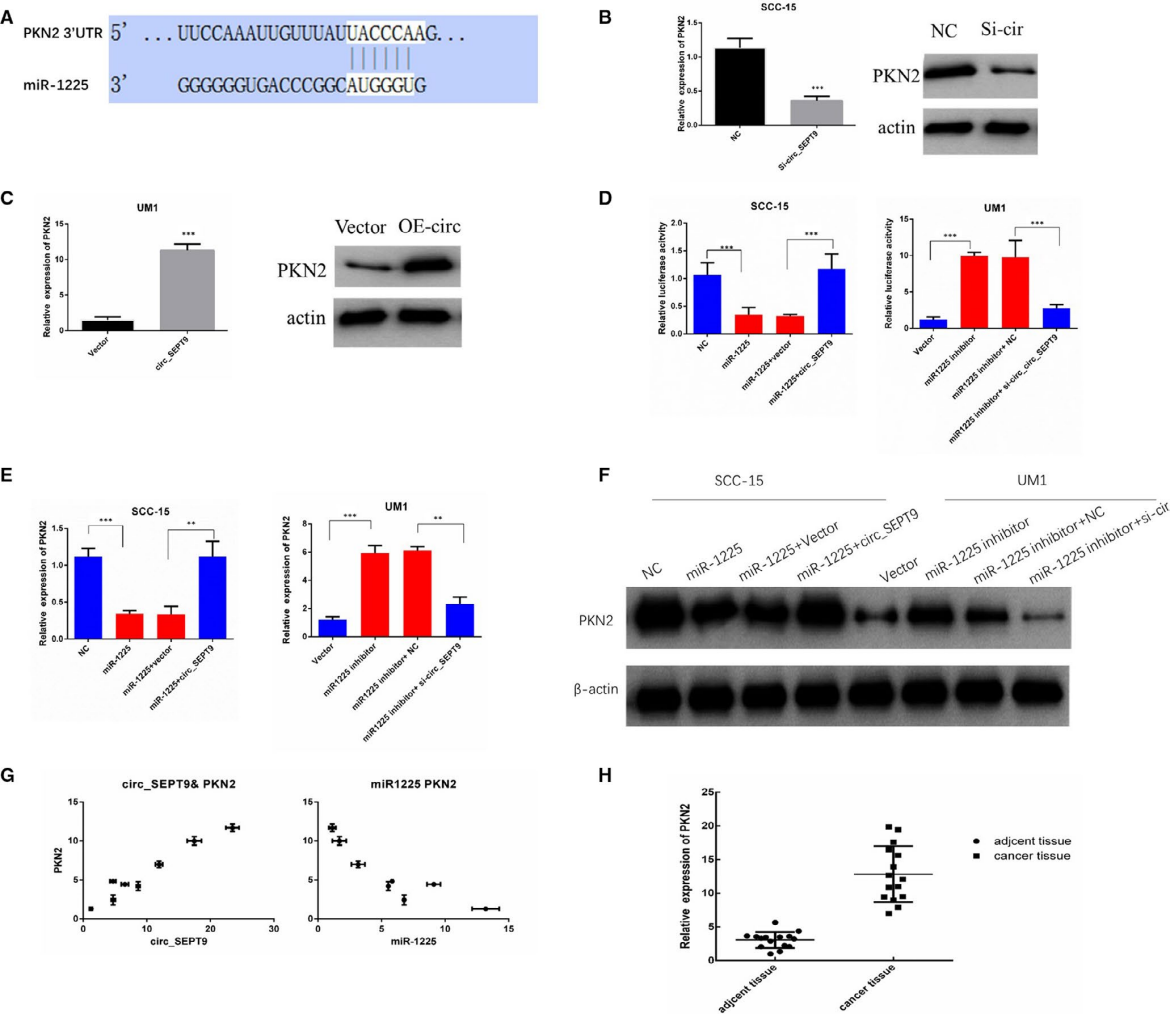
circ_SEPT9/miR‐1225 regulate the mRNA and protein level of PKN2. A, Diagram of predicted interactions between PKN2 mRNA and miR‐1225. B, qRT‐PCR and Western blot were used to detect the mRNA and protein level of PKN2 after silence of circ_SEPT9 in SCC‐15. C, qRT‐PCR and Western blot were used to detect the mRNA and protein level of PKN2 after overexpression of circ_SEPT9 in UM1. D, Luciferase activities of circ_SEPT9 in SCC‐15 and UM1 cells after indicated treatment. E, qRT‐PCR was used to detect the mRNA level of PKN2 in SCC‐15 and UM1 cells after indicated treatment. F, Western blot was used to detect the protein level of PKN2 in SCC‐15 and UM1 cells after indicated treatment. G, Positive correlation between circ_SEPT9 and PKN2 in OSCC specimens was shown (Right). Negative correlation between miR‐1225 and PKN2 in OSCC specimens was shown (Left). H, qRT‐PCR analysis for the expression of PKN2 in OSCC tissue and the adjacent normal tissue

One of the most common functions of microRNAs is to impair the stability of mRNA, and further influences the protein level of indicated gene. We applied the bioinformatics analysis to search for the possible target of miR‐1225. Interestingly, TargetScan predicted that PKN2 may be the potential target gene. Protein kinase N (PKN) is one member of protein kinase C (PKC). PKN family consists three members. Protein kinase N2 (PKN2) was the first one which was discovered at 1994.^15^ PKN2 is one of PKC‐related serine/threonine‐protein kinases and acts as Rho GTPase effector in various cellular signalling axis. The predicted binding site between miR‐1225 and PKN2 3’UTR is shown in Figure 6A.

The expression of PKN2 was detected by qRT‐PCR and Western blot. The results showed that silence of circ_SEPT9 down‐regulated both mRNA and protein level of PKN2 (Figure 6B), and transfection of circ_SEPT9 expression plasmids increased both mRNA and protein level of PKN2 (Figure 6C). All Western blot results have been quantified and analysed by ImageJ (Figure S1 C,D).

As circ_SEPT9 acted as sponge for miR‐1225, we next investigated whether circ_SEPT9 could rescue PKN2 expression impaired by miR‐1225. To verify whether PKN2 was the target of circ_SEPT9/miR‐1225 in OSCC cells, we performed the luciferase assay, qRT‐PCR and the Western blot in SCC‐15 and UM1 cells. Transfection of miR‐1225 could inhibited the activity of the PKN2 luciferase (Figure 6D), and the mRNA and protein level of PKN2 in SCC‐15 (Figure 6E,F). The influence of miR‐1225 on PKN2 was effectively rescued by transfection of circ_SEPT9 overexpression plasmids (Figure 6D‐F). miR1225 inhibitor enhanced the luciferase activity of PKN2, and increased PKN2 mRNA and protein level (Figure 6D‐F). Silence of circ_SEPT9 could partly block the influence of miR‐1225 inhibitor on PKN2 (Figure 6D‐F). All the Western blot results have been quantified and analysed by ImageJ (Figure S1 E,F).

Interestingly, PKN2 mRNA level was significantly higher in OSCC cells with high expression of circ_SEPT9 and low level of miR‐1225. The expression of PKN2 mRNA was positively correlated with circ_SEPT9 expression and negatively correlated with miR‐1225 expression（Figure 6G）. What's more, tumour tissue had more abundant expression of PKN2 than the compared adjacent normal tissue (Figure 6H). All the data confirmed that circ_SEPT9 regulated PKN2 expression through regulating miR‐1225 expression.

### 3.8 PKN2 promotes OSCC progression

**FIGURE 7:**
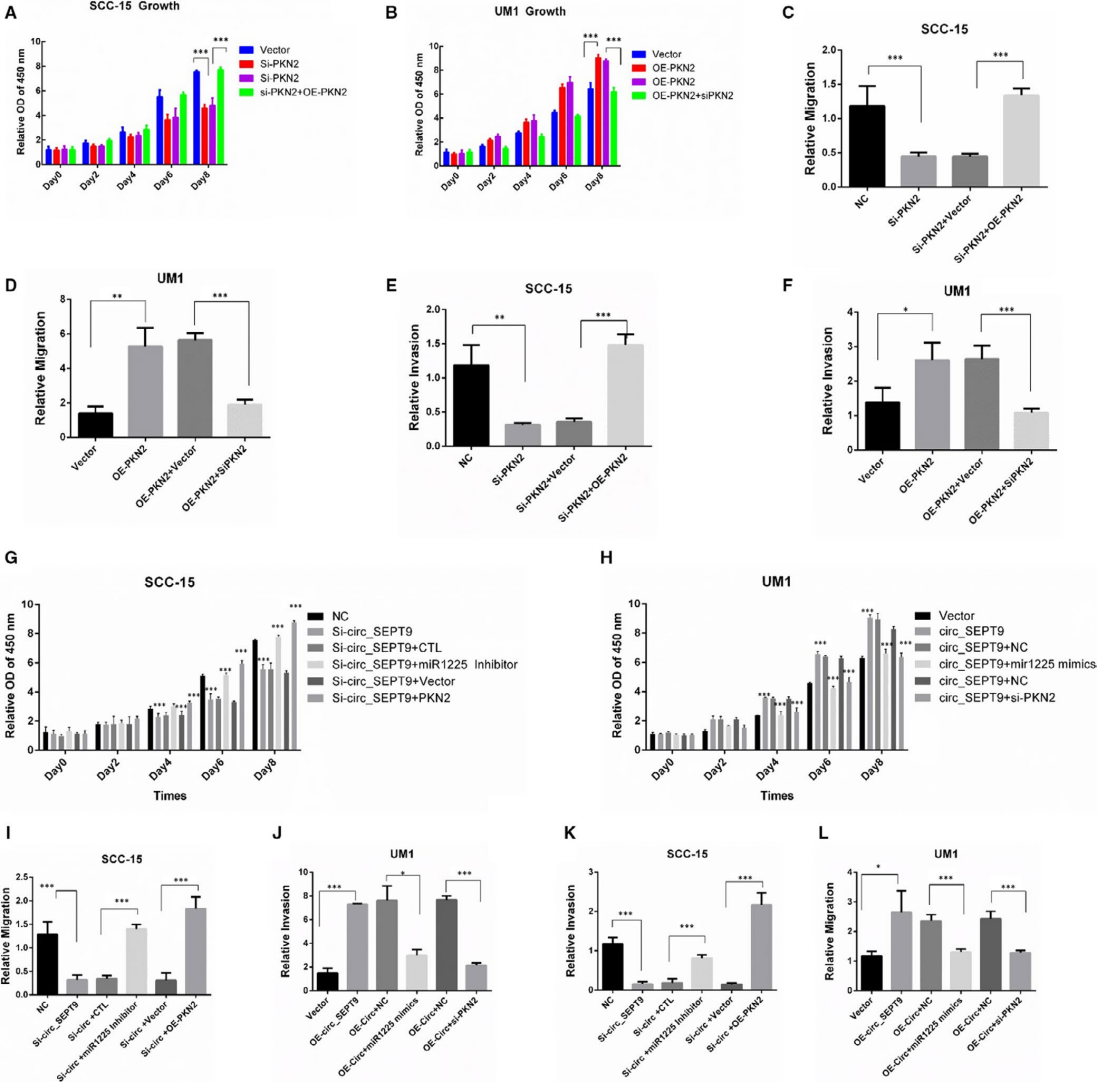
circ_SEPT9/miR‐1225/PKN2 contribute to the progression of OSCC cells. A, CCK8 was performed to detect the growth rate of SCC‐15. B, CCK8 was performed to detect the growth rate of UM1. C, Wound assay was performed in SCC‐15 cells. Photographs were taken by microscope at 0 and 24 h. D, Wound assay was performed in UM1 cells. Photographs were taken by microscope at 0 and 24 h. E, Transwell assay was performed in SCC‐15 cells. F, Transwell assay was performed in UM1 cells. G, SCC‐15 cells were transfected with indicated plasmids. CCK8 was performed to detect the growth rate. H, UM1 cells were transfected with indicated plasmids. CCK8 was performed to detect the growth rate. I, Wound assays were performed to detect the migration ability of SCC‐15. J, Wound assays were performed to detect the migration ability of UM1. K, Transwell assays were used to detect the invasion ability of SCC‐15. L, Transwell assays were used to detect the invasion ability of UM1

Previous studies showed that PKN2 was required for cell cycle, cell adhesion,^16^ transcription activation,^17^ tumour cell migration, invasion^18^ and apoptosis,^19^ indicating that PKN2 might play important roles in tumour progression. We investigated the functions of PKN2 in OSCC progression by CCK8, migration and invasion assays. The results showed silence of PKN2 could decrease the ability of proliferation, migration and invasion in SCC‐15 cell line, and re‐expression of PKN2 would rescue proliferation and metastasis effectively (Figure 7A,C,E). Overexpression of PKN2 enhanced the tumour growth, migration and invasion ability, while the ability significantly decreased in the presence of PKN2 siRNAs in UM1 cells (Figure 7B,D,F). The results confirmed that PKN2 promoted OSCC proliferation, migration and invasion.

### 3.9 circ_SEPT9/miR‐1225/PKN2 axis contributes to OSCC progression

To confirm the role of circ_SEPT9/miR‐1225/PKN2 axis in OSCC progression, we performed CCK8, migration and invasion assays. We silenced circ_SEPT9 in SCC‐15 cells and then transfected with miR‐1225 inhibitor or PKN2 overexpression plasmids. The results showed that inhibition of miR‐1225 or overexpression of PKN2 could effectively rescue circ_SEPT9 silence induced decrease in proliferation, migration and invasion (Figure 7G,I,K). We also transfected miR‐1225 mimics and PKN2 siRNAs into circ_SEPT9 overexpressed UM1 cells. Overexpression of miR‐1225 or silence of PKN2 could block circ_SEPT9 overexpression induced OSCC progression enhancement (Figure 7H,J,L).

**FIGURE 8:**
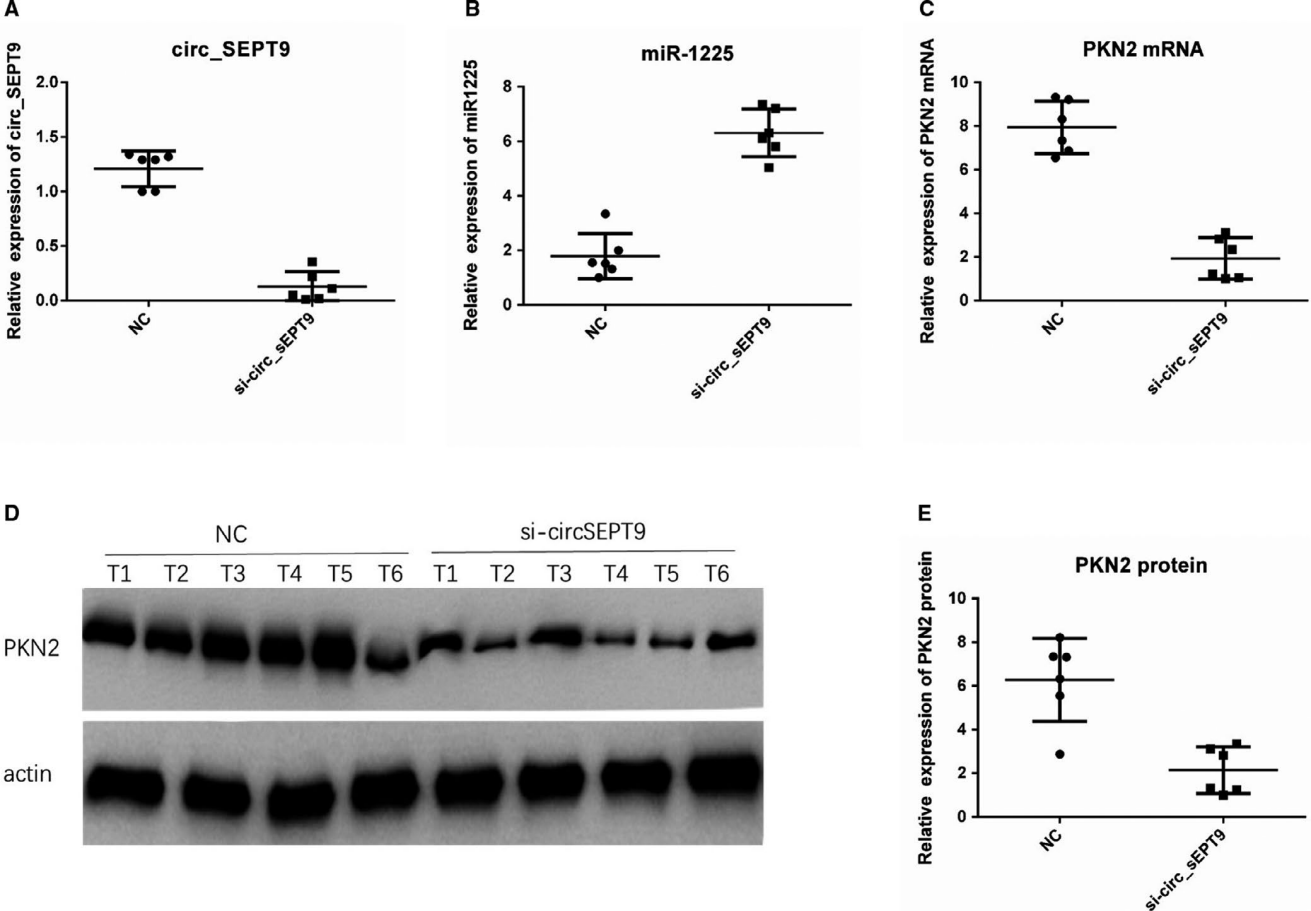
circ_SEPT9/miR‐1225/PKN2 axis were confirmed in vivo. A, qRT‐PCR was performed to detect the expression of circ_SEPT9 in tumours of nude mice. B, qRT‐PCR was performed to detect the expression of miR‐1225 in tumours of nude mice. C, qRT‐PCR was performed to detect the expression of PKN2 in tumours of nude mice. D, Western blot was performed to detect the expression of PKN2 in tumours of nude mice. E, Relative protein expression of PKN2 was calculated by ImageJ. β‐Actin was used as the loading control

We also detected circ_SEPT9/miR‐1225/PKN2 axis in the tumour of nude mice. The results showed that tumours with lower expression of circ_SEPT9 (Figure 8A) had higher level of miR‐1225 (Figure 8B), lower PKN2 mRNA (Figure 8C) and protein level (Figure 8D,F). The results confirmed existence of circ_SEPT9/miR‐1225/PKN2 axis in vivo.

Both in vitro and in vivo experiments confirmed that circ_SEPT9/miR‐1225/PKN2 axis contributed to OSCC progression.

## 4 DISCUSSION

Cancer progression is the main factor leading to 90% of the deaths in cancer patients. Uncovering the underlying mechanisms of cancer proliferation and metastasis is of great value for cancer therapy. In our research, we found that OSCC metastasis was associated circular RNA SEPT9, which was significantly up‐regulated in OSCC cancer cells. Stepwise investigations showed that circ_SEPT9 acted as sponge for miR‐1225 and protected PKN2 mRNA from degradation. More importantly, silence of circ_SEPT9 suppressed OSCC proliferation, migration and invasion significantly, indicating that circ_SEPT9 may be a promising therapeutic target for OSCC.

As a newly found non‐coding RNA, circular RNA has attracted lots of attention because of its stable loop structure. Due to improvements in high‐throughput sequencing technology and data analysis technique, study of circular RNAs has been more and more important and becomes a major area of non‐coding RNA research.^20^, ^21^

It has been reported that circular RNAs are aberrantly expressed in various disease and play critical important roles in progression of disease.^22^, ^23^, ^24^ However, their specific function remains largely undiscovered in cancer, including OSCC.

As non‐classical transcriptional products, circular RNAs are synthesized in the nucleus and mature in the cytoplasm.^25^, ^26^ circRNAs act as microRNA sponges by forming circRNA/miRNA complexes at specific binding sites.^27^ circRNAs are produced from pre‐mRNAs splicing events, which the exons or introns are covalently linked and form single‐stranded loops without 5' caps or a 3' poly‐A tails. Circular RNAs show more resistance to RNaseR than the linear RNAs due to the loop structure. Due to the stability of circular RNA, it has more time to play a role in biological activities than mRNA.^28^, ^29^, ^30^

circ_SEPT9 is transcripted from SEPT9 gene. Septins are a highly conserved proteins family consisting 14 members (SEPT1‐SEPT14).^31^ Septins are of great importance in cell activities such as proliferation and cell polarity.^32^ In our research, we found that circ_SEPT9 promoted the OSCC progression without influencing SEPT9 mRNA and protein expression. Our research discovered a novel mechanism which SEPT9 regulated OSCC progression.

More and more evidences have suggested the critical role of miRNAs in numerous biological processes, including cell cycle, metastatic, metabolism and apoptosis. miRNAs provide feedback by regulating several targets in the complex networks.^33^ miR‐1225 suppresses progression in several cancers.^34^ For example, microRNA‐1225 inhibited pancreatic cancer cell apoptosis via targeting JAK1.^35^ Our research revealed that miR‐1225 could be regulated by circ_SEPT9 through acting as microRNA sponge in OSCC cells. The research provided us a new mechanism about microRNA regulation which would be very important for non‐coding RNA network researches.

In our research, we focus on miR‐1225 which have been predicated to have binding site with circ_SEPT9 by bioinformatics analysis. RNA immunoprecipitation confirmed the directly interaction between circ_SEPT9 and miR‐1225 in vitro. Both silence and overexpression of circ_SEPT9 significantly affected miR‐1225 expression. Consistence with this, miR‐1225 was highly expressed in normal adjacent tissue than cancer tissue. Expression of miR‐1225 and circ_SEPT9 is negatively correlated in OSCC. All the data indicated that miR‐1225 was the target of circ_SEPT9 in OSCC. Functional experiments showed that overexpression of miR‐1225 greatly suppressed OSCC cell proliferation, migration and invasion.

Protein kinase N family (PKN) is one member of protein kinase C family (PKC) which consists of three isoforms, PKN1, PKN2 and PKN3.^36^, ^37^, ^38^ PKN2 was discovered at 1994 by Parker PJ et al^39^ PKN2 is a serine/threonine‐protein kinase and acts as effector of Rho GTPase in various cellular processes. It has been reported that PKN2 is essential in proliferation, metastasis, adhesion and gene transcription.^36^, ^38^, ^40^ In our research, we found that miR‐1225 could target PKN2 in OSCC cells. Overexpression of miR‐1225 decreased PKN2 mRNA and protein level which could be rescued by transfection of circ_SEPT9. The results confirmed that PKN2 was the target gene of miR‐1225 which could be regulated by circ_SEPT9. Through further experiments, we confirmed that PKN2 was responsible for circ_SEPT9 induced OSCC proliferation, migration and invasion. Our research has discovered a novel mechanism for PKN2 regulation which would provide a new thought about targeted therapy for tumour progression.

In the research, we found that circ_SEPT9 promoted OSCC proliferation and metastasis by regulating miR‐1225 and PKN2 levels. What's more, overexpression of miR‐1225 or silence of PKN2 completely blocked OSCC progression enhanced by circ_SEPT9. Collectively, our results strongly confirmed that circ_SEPT9 regulated miR1225/PKN2 axis by acting as a competing endogenous RNA. circ_SEPT9/miR‐1225/PKN2 signalling pathway is of great value for OSCC targeted therapy.

## CONFLICT OF INTEREST

All authors declared no potential conflicts of interest.

## AUTHOR CONTRIBUTIONS

**Chen Zou:** Funding acquisition (lead); Resources (lead). **Yilong Ai:** Conceptualization (equal); Data curation (equal); Resources (equal); Software (equal). **Zhe Tang:** Conceptualization (equal); Resources (equal). **Haigang Wei:** Methodology (supporting); Writing‐original draft (supporting). **Siyuan Wu:** Project administration (supporting); Writing‐review & editing (supporting). **Dahong Huang:** Investigation (lead); Validation (equal).

## ETHICAL APPROVAL

Ethical approval was obtained from the ethics committee of Foshan Stomatological Hospital.

## STATEMENT OF ANIMAL RIGHTS

All animal experiments were approved by the Animal Policy and Welfare Committee of School of Stomatology and Medicine, Foshan University.

## STATEMENT OF CONSENT

Written informed consent was obtained from all the patients.

## Supporting information


**Fig S1.** A‐B. Relative protein expressions of SEPT9 were calculated by Image J. β‐actin was used as the loading control. C‐D. Relative protein expressions of PKN2 were calculated by Image J. β‐actin was used as the loading control. E‐F. Relative protein expressions of PKN2 were calculated by Image J. β‐actin was used as the loading control.

## Data Availability

The data that support the findings of this study are available from the corresponding author upon reasonable request.
