# Supplementary figures and images for: circ_SEPT9, a newly identified circular RNA, promotes oral squamous cell carcinoma progression through miR‐1225/PKN2 axis
Source: J Cell Mol Med. 2020 Oct 14;24(22):13266–77. doi: 10.1111/jcmm.15943 (PMC7701517; doi:10.1111/jcmm.15943)

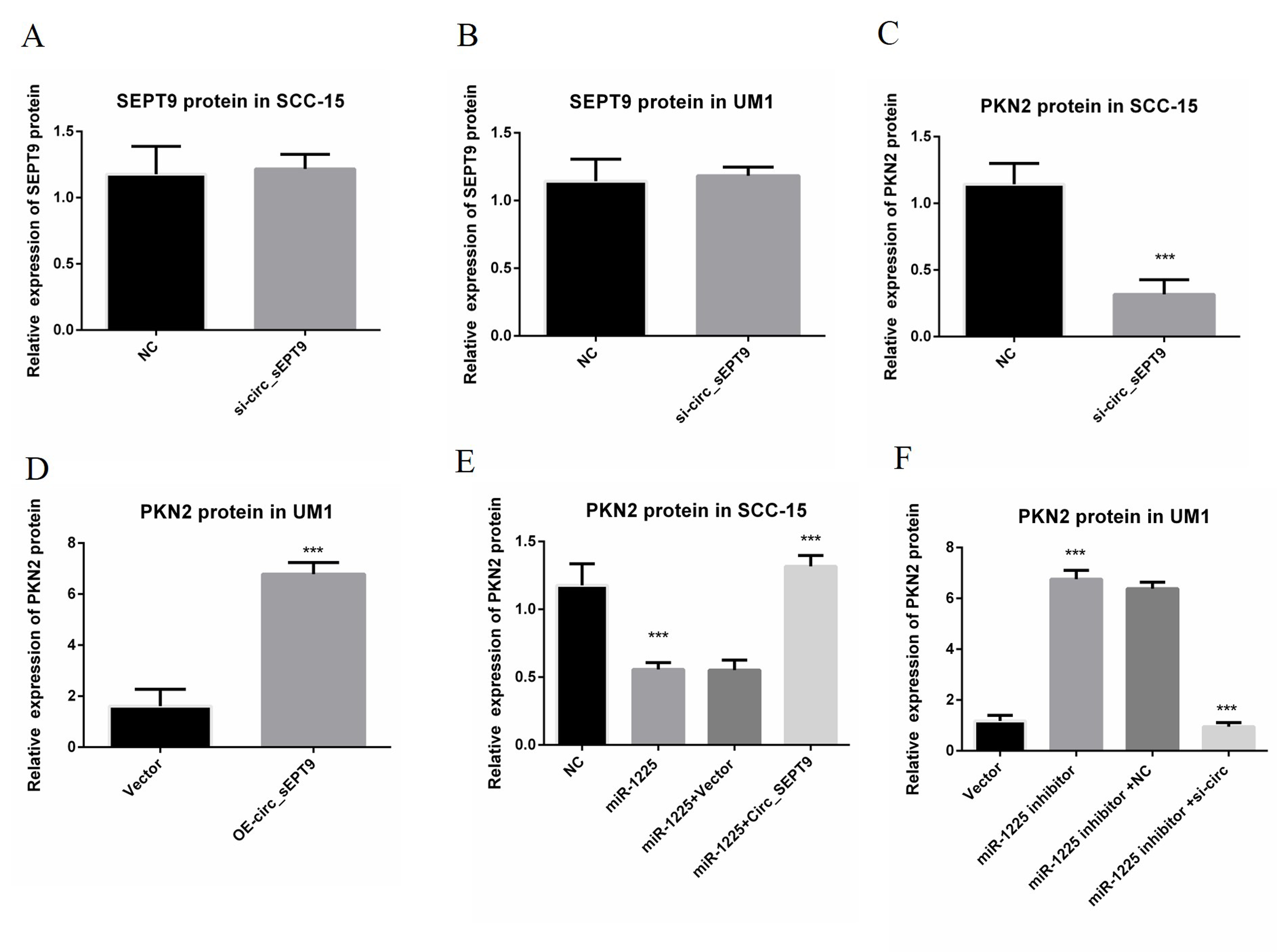

Supplement: Supplementary file 1 — Fig S1. A‐B. Relative protein expressions of SEPT9 were calculated by Image J. β‐actin was used as the loading control. C‐D. Relative protein expressions of PKN2 were calculated by Image J. β‐actin was used as the loading control. E‐F. Relative protein expressions of PKN2 were calculated by Image J. β‐actin was used as the loading control. [file JCMM-24-13266-s001.tif]
